# Tanshinone I Enhances the Pulmonary Immune Response of CD8^+^ T Cells by Promoting Memory Differentiation

**DOI:** 10.3390/biomedicines13112805

**Published:** 2025-11-18

**Authors:** Manqiu Wang, Honglei Wang, Yaling Wang, Changxing Gao, Leqi Fan, Jing Li, Qing Zhu

**Affiliations:** State Key Laboratory of Bioactive Substance and Function of Natural Medicines, Chinese Academy of Medical Sciences and Peking Union Medical College Institute of Materia Medica, Beijing 100050, China; wangmanqiu@imm.ac.cn (M.W.); wanghonglei@imm.ac.cn (H.W.); wangyaling@imm.ac.cn (Y.W.); gaochangxing@imm.ac.cn (C.G.); fanleqi@imm.ac.cn (L.F.); lijing_jeanette@imm.ac.cn (J.L.)

**Keywords:** tanshinone I, vaccination, CD8^+^ T cell, central memory, effector memory, lung Trm, mucosal immunity

## Abstract

**Objectives:** Vaccination by a nonmucosal route to elicit CD8^+^ T cell-mediated mucosal immunity against respiratory infections presents a great challenge for the development of an effective vaccine or immunization strategy. This study aimed to explore a new strategy to address the challenge. **Methods:** To test this strategy, s.c. vaccinated mice were administered i.p. with tanshinone I (TSN1), a main bioactive compound found in the root of *Salvia miltiorrhiza*, and CD8^+^ T cell responses were analyzed using flow cytometry. The differentiating effects of TSN1 on CD8^+^ T cells from naïve mice were also evaluated in an in vitro setting. **Results:** Nonmucosal vaccination and administration of TSN1 induce pulmonary-resident vaccine-specific memory CD8^+^ T cells through increased lung-specific recruitment and retention. The improved memory response appears to result from the impact of TSN1 introduced during the primary immunization phase. Given a specific range of varying concentrations of this natural compound, it exhibits a differential effect on the memory differentiation of CD8^+^ T cells in the process of being activated. Effector memory T cells expand robustly relative to central memory T cells, and both memory subsets have additionally increased expression of CD44 and CD69. With more potent cytolytic activity, CD8^+^ Trm expressing CD69 particularly predominate the population lacking the CD69 expression in the lungs of TSN1-treated mice. **Conclusions:** Our study suggests that TSN1 as an important natural compound may hold great promise for novel approaches to the design and development of a more practical and efficient vaccination strategy to generate effective respiratory mucosal immunity.

## 1. Introduction

CD8^+^ T cell-mediated immunity is crucial in protecting against respiratory infection caused by highly mutated viruses [1]. Vaccination against a pathogen-specific antigen is capable of eliciting a cellular response to the antigen by CD8^+^ T cells, which are central to the formation and sustenance of the immunological memory [2,3,4,5,6]. Long-lived memory CD8^+^ T cells retain robust proliferative and effector capacities and are poised to respond swiftly upon re-encountering the same pathogen [7]. They exist as heterogeneous populations that can be practically subdivided into effector memory (Tem) cells and central memory (Tcm), which circulate through the lymphoid and peripheral tissues, respectively, and tissue-resident memory (Trm) cells, which become established in the peripheral and lymphoid tissue as noncirculating cells [8,9,10,11]. These subsets constitute the memory cell repertoire that contains numerous memory clones which are maintained in and segregated by tissue as a location-specific reservoir, retaining the capacity for extensive clonal expansion [12,13].

Respiratory infections, such as those caused by the corona, influenza, and respiratory syncytial viruses, which are limited to the airways, can be easily transmitted to others from asymptomatic carriers. Local mucosal immunity is thus required to more effectively control and prevent the spread of such infections [14,15]. However, novel vaccines expected to elicit mucosal immunity are often first developed for vaccination via a nonmucosal route, which is more important for the protection against systemic infection [1,16,17]. As a result, several years could elapse before an ideal mucosal vaccine formulation becomes available. Based on the fact that mucosal vaccines for respiratory infections remain few and far between, identification of novel agents to offer nonmucosal vaccines tailored to the needs of mounting potent respiratory mucosal immunity, especially lung CD8^+^ Trm, is highly desirable. The generation and persistence of lung Trm appears to rely on constant replenishment from recirculating Tem [18], which can be derived from Tcm [19,20]. Therefore, the sufficient formation of Tem and Tcm may be crucial for the establishment of Trm in the lung.

A growing body of research suggests that natural products, derived from plants as well as other sources, possess immunomodulatory properties, and certain natural compounds are found to enhance CD8^+^ T cell responses [21,22,23]. Some immunomodulatory phytochemicals have been shown to be capable of regulating CD8^+^ T cell memory differentiation. Metformin has the ability to increase the number of central memory and stem-like memory CD8^+^ T cells while simultaneously suppressing effector T cells [24]. This is in contrast to a metformin derivative, which favors the differentiation of short-lived effectors that are specifically for viruses [25]. A more recent approach demonstrates the role of berberine in improving the formation of CD8^+^ Tcm, which correlates with greater systemic protection against microbial infection, but substantially inhibits the expansion of Tem [26]. In another study, dichloromethane extracts from *Teucrium montanum* were found to be able to drive CD8^+^ proliferation while promoting Tcm formation [27]. Although natural products have shown some promising effects in modulating T cell memory differentiation, they are still underexplored for the design of novel vaccines and the development of vaccination or immunotherapy strategies.

Tanshinone I (TSN1), an abietane diterpenoid, is one of the important natural components found primarily in the root of *Salvia miltiorrhiza* (commonly known as Danshen). It has been used for anti-inflammation, anticancer, and cardiovascular protection due to its therapeutic potential as well as minimal cytotoxicity and genotoxicity [28,29,30]. Recent studies indicate that TSN1 exerts antiviral effects [31,32]. In the present study, we evaluated the immunomodulatory role of TSN1 in the memory differentiation of activated CD8^+^ T cells, and sought to determine whether TSN1 could help increase the formation of CD8^+^ Tem and Tcm and whether regular nonmucosal vaccination with concurrent use of TSN1 would facilitate the host generating respiratory CD8^+^ Trm and their functionality.

## 2. Materials and Methods

### 2.1. Animals

C57BL/6 CD45.2 mice, 6–8 weeks of age, were obtained from Beijing Vital River Laboratory Animal Technology (a subsidiary of Charles River Laboratories International, Beijing, China). They were housed at 23 ± 3 °C temperature and 55 ± 15% relative humidity with a 12 h light/dark cycle in specific pathogen-free facilities. Food and water were provided ad libitum to the animals. The research was conducted in accordance with all institutional guidelines and ethics and approved by the Laboratories Institutional Animal Care and Use Committee of the Chinese Academy of Medical Sciences and Peking Union Medical College. All experimental protocols were approved by the Institute of Materia Medica Animal Authorities (Beijing, China).

### 2.2. Reagents

**Figure 1:**
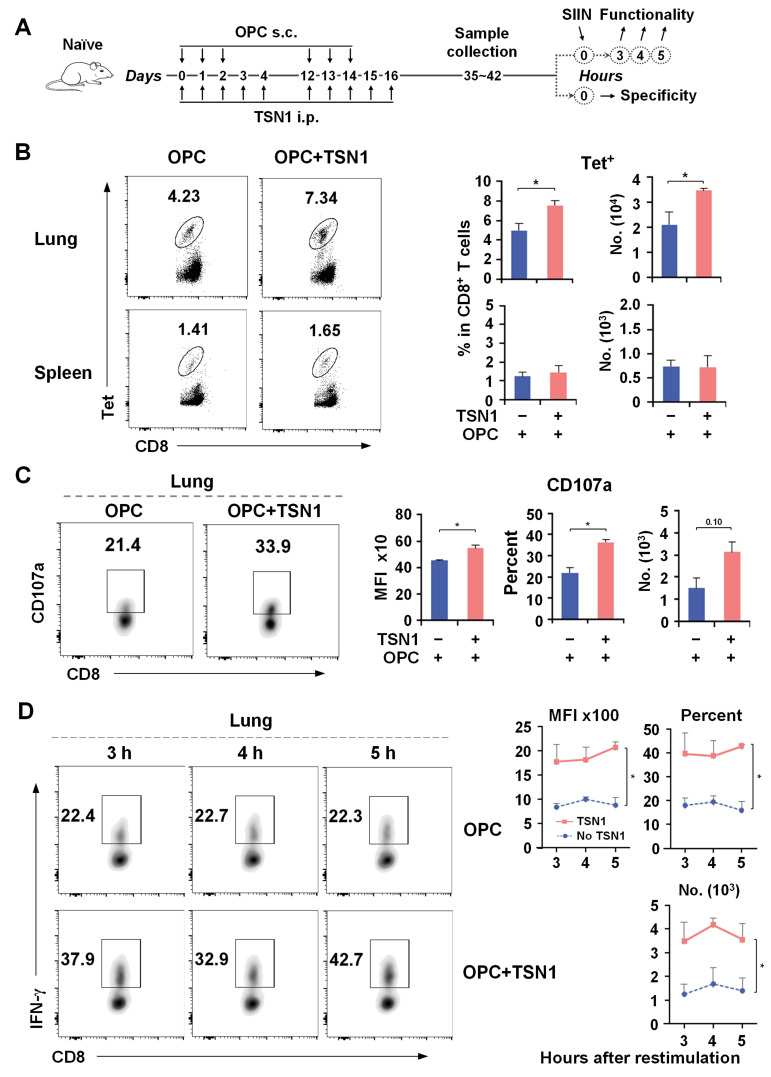
CD8^+^ T cells activated with TSN1 have a greater cytolytic potential in the lung. Mice were immunized s.c. by footpad injection of the combination of OVA protein, poly(I:C), and CpG (OPC) while receiving 10 mg/kg bw/d of TSN1 given i.p. followed by a s.c. boost (prime-boost mice). Cells were isolated from the lungs and spleen at d35–42 after the first vaccination and examined for antigen-specific T cells by flow cytometry. Tet^+^ CD8^+^ T cells were gated for analysis. (**A**) Schematic representation of the immunization regimen used to induce an OVA-specific T cell response and sample collection. (**B**) Percentage and number of Tet^+^ cells in CD8^+^ T cells in the lung and spleen. (**C**) Mean fluorescence intensity (MFI) of CD107a expression and percentage and number of CD107a^+^ cells in Tet^+^CD8^+^ T cells in the lung after antigen restimulation. (**D**) MFI of IFN-γ expression and percentage and number of IFN-γ^+^ cells in Tet^+^CD8^+^ T cells after 3, 4, and 5 h of restimulation. Results are expressed as mean ± SEM (*n* = 4 per group). One-tailed Mann–Whitney test was used for statistical analysis. * *p* < 0.05 indicates a significant difference between groups.

Poly(I:C) was purchased from InvivoGen (San Diego, CA, USA). CpG 1826 was synthesized by the Chinese Peptide Company (Hangzhou, China). All were free of endotoxin and protein contamination. OVA and SIINFEKL were bought from Sigma-Aldrich (St. Louis, MO, USA) and Solarbio (Beijing, China), respectively. TSN1 (Figure 1A) was obtained from Yuanye (Shanghai, China). The MHC tetramers (tet) loaded with TetOVA_257–264_ SIINFEKL or TetPA_224–233_ SSLENFRAYV peptides were obtained from the NIH Tetramer Core Facility (Atlanta, GA, USA). Antibodies for flow cytometry were all purchased from Thermo Fisher Scientific (Waltham, MA, USA) or BioLegend (San Diego, CA, USA) unless specified elsewhere.

### 2.3. Vaccination

To induce an OVA-specific response, mice (*n* = 3–6) were vaccinated by s.c. injection at the footpad with a mixture of 5 μg OVA, 30 μg poly(I:C), and 3 μg CpG1826 ODNs (OPC) in a volume of 50 μL daily for 3 days (OPC mice). To induce a PA-specific response, a mixture of PA_224–233_ and OVA, instead of OVA only, was given together with poly(I:C) and CpG (PA + PC) (PAPC mice, *n* = 3). TSN1 was administered intraperitoneally (i.p.) to these mice at daily doses of 20 mg/kg body weight (mg/kg bw/d) for 5 days from the first day of vaccination. To strengthen the body’s initial response, one group of OPC mice was boosted with the same vaccine formulation on days 12, 13, and 14 (prime-boost mice) and assessed for the antigen-specific T cell response. The other group of OPC mice was inoculated with 100 μg OVA daily in two weeks via aerosol inhalation (a.i.) instillation, while PAPC mice were given 100 μg PA_224–233_ instead. Bioassays were performed on the indicated days.

### 2.4. Intravenous Labeling of T Cells and Lymphocyte Isolation

Intravascular T cells were labeled in vivo by i.v. injection of 3 μg of anti-CD45 antibody. Mice were euthanized at 3 min postinjection, followed by bronchoalveolar lavage and lung perfusion with ice-cold PBS. Lymphocytes were isolated by treatment of minced lungs with collagenase IV (Thermo Fisher Scientific, Waltham, MA, USA).

### 2.5. In Vitro Activation of Lymphocytes and Antigen Restimulation

Splenocytes were isolated from wild-type mice and incubated with ACK lysis buffer for 5 min to remove erythrocytes. Cells were cultured in RPMI-1640 supplemented with 10% fetal bovine serum, 100 U/mL penicillin, 100 mg/mL streptomycin, and 100 U/mL IL-2 (Peprotech, Cranbury, NJ, USA) at 37 °C in 5% CO_2_. These cells were stimulated with 5 mg/mL anti-CD3 and 2.5 mg/mL anti-CD28 mAbs (145-2C11 and 37.51, respectively; BioLegend, San Diego, CA, USA) in the presence of TSN1 for 3 days. Dose-dependent effects of TSN1 were assessed at concentrations ranging from 0.02 to 15 μM. For further analysis, 1 and 5 μM found to be the Tem- and Tcm-specific concentrations, respectively, were used. Cell counts for each subset were normalized to the corresponding subset of the α3/28 control, and then scaled by a factor of 10 to express the data in a convenient range that facilitates data visualization and allows for interpretable comparisons.

To examine the CD8^+^ T cell response, freshly isolated cells from immune mice were rested overnight before they were seeded at a density of 2 × 10^6^ per well in 96-well plates for restimulation with SIINFEKL peptides at 100 nM in the presence of 1 μg/mL of brefeldin A at 37 °C.

### 2.6. Flow Cytometry

Prior to antibody staining, FcγIII/II receptors were blocked by incubating cells with anti-CD16/32 (2.4G2; CyTek Biosciences, Fremont, CA, USA). Fluorescent dye-labeled anti-CD107a antibody was added at the beginning of antigen stimulation. Cells were collected at the indicated times of the stimulation, and surface stained with antibodies for CD8 (53-6.7), CD44 (IM7), CD62L (MEL-14), and CD69 (H1.2F3). Anti-CD107a (1D4B) mAbs were present from the beginning of the peptide stimulation for a period of 3, 4, or 5 h. For the detection of OVA-specific CD8^+^ T cells, cells were incubated with H-2Kb-SIINFEKL tetramers obtained from NIH Tetramer Core Facility for 23 min at 37 °C. Cells were then stained with other antibodies for an additional 22 min at 37 °C. After washing, cells were resuspended in PBS for flow cytometry analysis. For intracellular staining of IFN-γ (XMG1.2), cells were fixed in 4% paraformaldehyde, and permeabilized with 0.1% saponin before 30 min incubation with fluorescently labeled antibodies. All samples were acquired using a Urit BF-730 flow cytometer (Urit Medical Electronic, Guilin, China) or FACSVerse (Becton Dickinson, Franklin Lakes, NJ, USA) and analyzed with FlowJo software (10.8 TreeStar, Ashland, OR, USA). The percentage of positive cells for each marker or subset is indicated within the gates of the representative plots.

### 2.7. Statistics

Statistical analyses were performed using GraphPad Prism 9 software (Boston, MA, USA). The half-maximum effective concentration (EC_50_) and half-maximum inhibitory concentration (IC_50_) were determined by fitting the bell-shaped dose–response data using nonlinear regression analysis. Comparisons between groups were analyzed by one- or two-tailed Mann–Whitney test. Comparisons among means of more than two groups were determined by Kruskal–Wallis test. The *p*-values less than 0.05 were considered statistically significant.

## 3. Results

### 3.1. Treatment with TSN1 During Immunization Dictates the Capacity of CD8^+^ T Cells for Lung Homing

We first sought to determine whether TSN1 administration during the systemic (nonmucosal) vaccination would be sufficient to induce a more potent pulmonary memory CD8^+^ T cell response than vaccination only (no TSN1). To test this, we vaccinated mice s.c. daily for a duration of 3 days with a mixture of OVA protein, poly(I:C), and CpG (OPC), and administered 10 mg/kg bw/d of TSN1 i.p. (also systemically) to the mice (OPC + TSN1) from the first vaccination day daily for a duration of 5 days (i.e., two additional days of TSN1 with no vaccination), followed by s.c. boosting in 14 days (Figure 1A). By the use of SIINFEKL tetramers at d35–42, tet^+^ OVA_257–264_-specific CD8^+^ T cells were evaluated. A higher frequency of lung Tet^+^ CD8^+^ T cells was detected in OPC + TSN1 mice than in OPC-only mice, whereas no significant difference between the groups of mice in the spleen was found (Figure 1B). Compared to boost with OPC only, TSN1 mice displayed a higher percentage of lung Tet^+^ CD8^+^ T cells that exhibited CD107a degranulation and IFN-γ production upon restimulation with OVA antigen (Figure 1C,D). The results indicated that the inclusion of TSN1 nonmucosally during vaccination could enhance the nonmucosal vaccine-induced cellular response in the lung, leaving us to speculate that TSN1 is more likely to have an impact on the memory differentiation of T cells.

### 3.2. TSN1 Mediates Biphasic Regulation of the Memory Differentiation of T Cells

**Figure 2:**
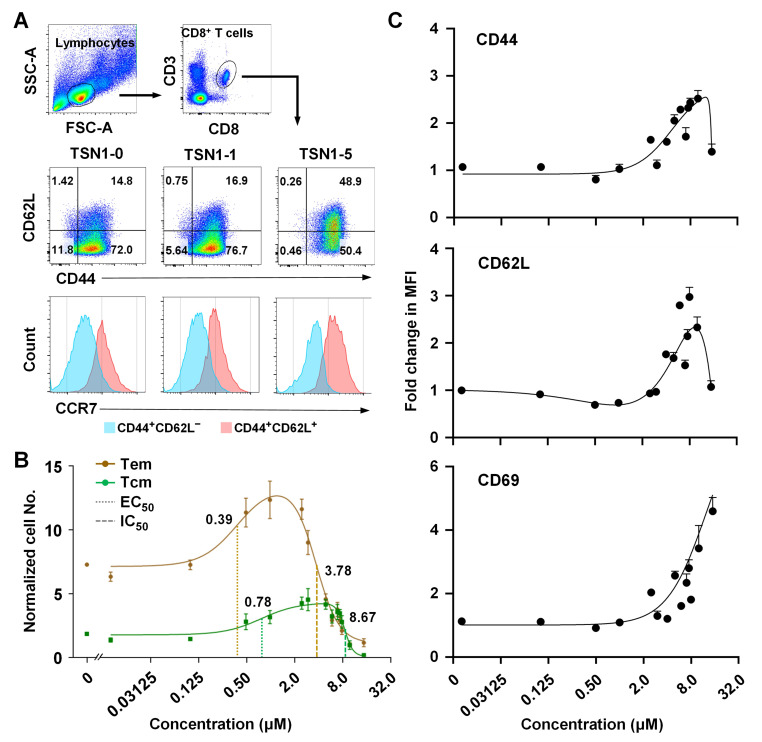
TSN1 has both positive and negative effects on Tem and Tcm differentiation of T cells. Splenocytes in equal numbers (2 × 10^5^) isolated from naïve mice were stimulated with anti-CD3 and anti-CD28 (α3/28) in combination of TSN1 for 3 days, followed by flow cytometry analysis. (**A**) Flow cytometry showing CD44 and CD62L (pseudocolor plots) and CCR7 (histograms) expression in CD8^+^ T cells. (**B**) Dose–response curves between TSN1 concentration and normalized number of Tcm (CD44^+^CD62L^+^) or Tem (CD44^+^CD62L^−^). The normalized cell number is obtained by dividing the α3/28 control value and then multiplying by 10. (**C**) Dose response of CD44, CD62L, and CD69 expression to TSN1 stimulation. Results are expressed as mean ± SEM (*n* = 3–4 per group). EC_50_/IC_50_: half-maximum effective/inhibitory concentration.

To understand how TSN1 contributes to CD8^+^ T cell memory differentiation and their peripheral tissue tropism, we next examined the dose–response curves for the association between TSN1 concentration and the number of Tem and Tcm subsets. Notably, the compound differentially promoted the formation of both Tem (CD44^+^CD62L^−^CCR7^−^) and Tcm (CD44^+^CD62L^+^CCR7^+^) subsets (Figure 2A). Lower concentrations, ranging from 0.1 to 1 μM, induced Tem, whereas a higher concentration range of 0.5 to 5 μM was required to promote Tcm, indicating the concentration-dependent biphasic effects of TSN1 (Figure 2B). While a significant increase in the normalized number of Tem was found at the concentrations of 0.5 and 1.0 μM (*p* < 0.05 and <0.01, respectively), the increase in Tcm numbers was more prominent at concentrations as high as 1.0–7.0 μM (*p* < 0.05–0.001). Beyond the maximum response (approximately 1.14 μM for Tem and 7.15 μM for Tcm, respectively), a dose-dependent inhibition of cell expansion was observed in both subsets responding to the compound (Figure 2B). In contrast to 1 μM of TSN1 (TSN1-1) which gave a considerably high ratio of Tem to Tcm (12.4:3.2), TSN1 at 5 μM (TSN1-5), where both Tcm increased and Tem decreased statistically significantly compared to control (*p* < 0.001 and <0.05, respectively), led to a decreased Tem:Tcm ratio (4.6:4.2, *p* < 0.001). Further upregulation of CD44, CD62L, and CD69 expression was seen for higher concentrations, mainly above 5 μM, suggesting an enhanced memory differentiation of T cells.

### 3.3. TSN1’s Differential Effect May Help Improve the Formation of Tem and Tcm

**Figure 3:**
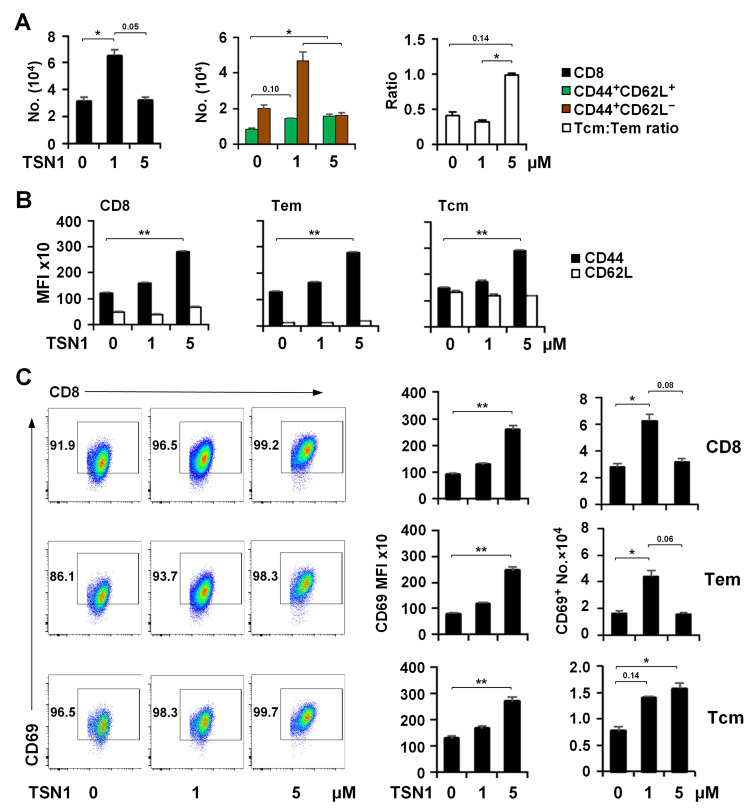
TSN1 drives the expansion of both Tem and Tcm at a lower concentration and enhances Tcm differentiation at a higher concentration. Splenocytes isolated from naïve mice were activated in the presence of 1 μM or 5 μM of TSN1 for 3 days before flow cytometry analysis. (**A**) Flow cytometry analysis of CD8^+^ T cells, Tcm, and Tem in number, and the ratio of Tcm to Tem. (**B**) MFI of CD44 and CD62L expression in CD8^+^ T cells, Tcm, and Tem. (**C**) MFI of CD69 in CD8^+^ T cells, Tcm, and Tem, and the number of CD69^+^ CD8^+^ T cells, Tcm, and Tem in CD8^+^ T cells. Results are expressed as mean ± SEM (*n* = 3 per group). Kruskal–Wallis test was used for statistical analysis. * *p* < 0.05 and ** *p* < 0.01 indicate a significant difference between groups.

Based on TSN1’s dose-dependent differential effects on CD8^+^ T cell differentiation, we opted to focus our further analysis on TSN1-1 and TSN1-5 owing to their profound impacts. TSN1-1 stimulated total CD8^+^ T cells and Tem to expand by over 2-fold in contrast to TSN1-5, which curbed their proliferation (Figure 3A). TSN1-1 could slightly promote Tcm to expand, while TSN1-5 increased the Tcm number significantly but to a much lesser extent compared to TSN1-1-mediated Tem expansion. As a result, TSN1 left the ratio of Tcm to Tem virtually unchanged at 1 μM in comparison to the no-treatment control, but brought the ratio of Tcm-to-Tem close to unity at 5 μM without affecting the total number of CD8^+^ T cells (Figure 3A). The difference in the number of Tcm between TSN1-5 and TSN1-1 was, however, not statistically significant (*p* = 0.10), suggesting a moderate effect of higher concentrations of TSN1 in increasing the amount of Tcm. Furthermore, in the presence of TSN1-1, neither the expression level of CD44 and CD62L (Figure 3B) nor CD69 (Figure 3C) was additionally upregulated. However, TSN1-5-modulated CD8^+^ T cells and their subsets Tem and Tcm displayed elevated expression of both CD44 and CD69 albeit with a minimal change in CD62L expression. The results confirmed that both Tem and Tcm could be effectively induced by TSN1, to which CD8^+^ T cells appeared to be more sensitive in Tem differentiation and potentially have a greater capacity to transmigrate from the blood circulation to the peripheral tissue.

### 3.4. TSN1 Facilitates the Recruitment and Retention of Memory CD8^+^ T Cells in the Lung

**Figure 4:**
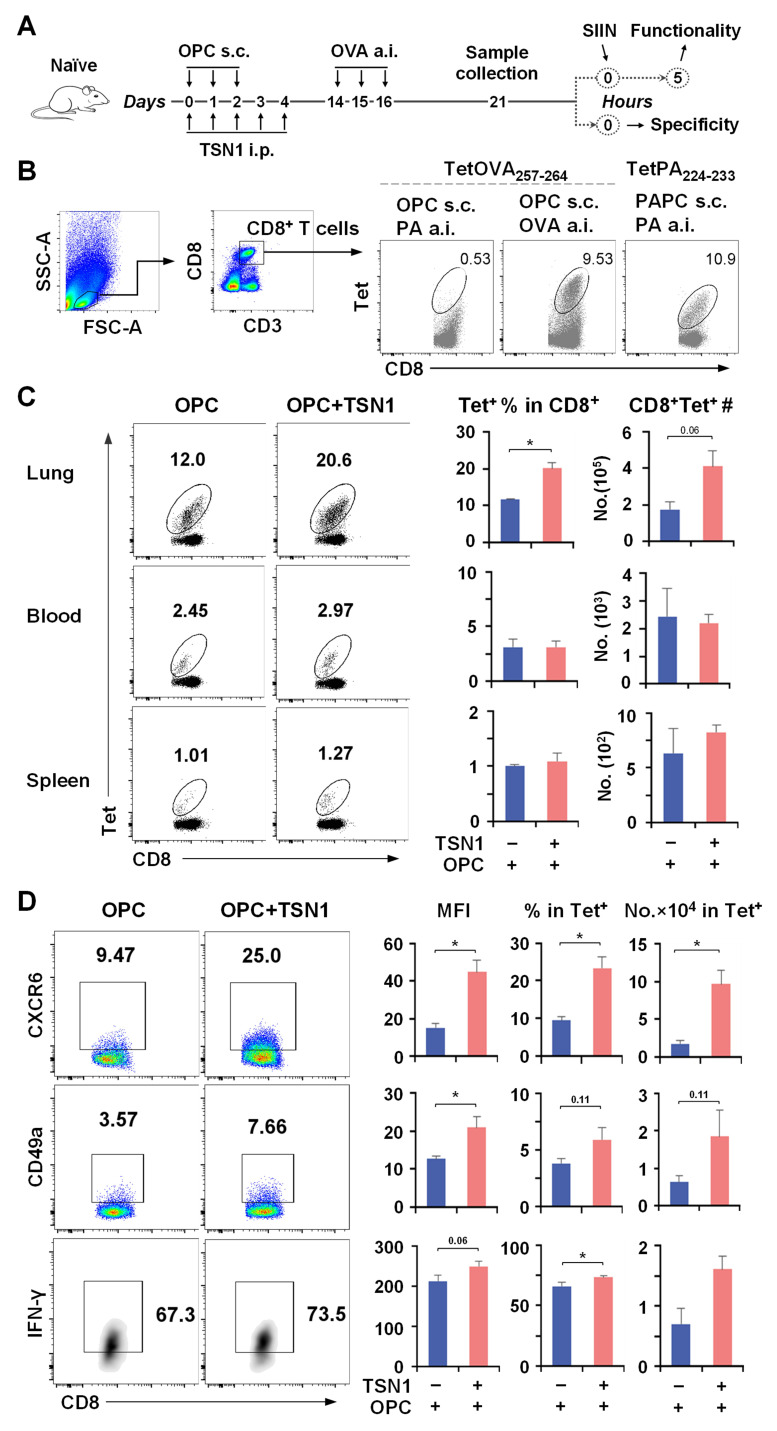
Subcutaneous immunization in combination with TSN1 induces CD8^+^ T cells with enhanced lung-specific homing capability. Mice were immunized s.c. by footpad injection of OPC or the combination of OVA with an irrelevant antigen PA_224–233_, poly(I:C), and CpG (PAPC) while receiving 10 mg/kg bw/d of TSN1 given i.p. (*n* = 3–4). The OVA or PA peptide was delivered by a.i. on d14. Cells were isolated from the lung, blood, and spleen at d21 after the vaccination and examined for antigen-specific T cells by flow cytometry. Tet^+^ CD8^+^ T cells were gated for analysis. (**A**) Schematic representation of the immunization regimen. (**B**) Percentage of TetOVA_247–264_^+^ or TetPA_224–233_^+^ cells in CD8^+^ T cells from the lung post a.i. (**C**) Percentage and number of Tet^+^ cells in CD8^+^ T cells from the lung, blood, and spleen. (**D**) MFI of CXCR6, CD49a, and IFN-γ, and percentage and number of CXCR6^+^, CD49a^+^, IFN-γ^+^ cells in Tet^+^CD8^+^ T cells from the lung. Results are expressed as mean ± SEM (*n* = 3–4 per group). One-tailed Mann–Whitney test was used for statistical analysis. * *p* < 0.05 indicates a significant difference between groups.

It is possible that the ability of vaccine-induced T cells to be mobilized to the lung could develop as early as after the primary vaccination when they were exposed to TSN1. We then assessed the TSN1-modulated pulmonary response of CD8^+^ T cells with regard to their antigen specificity and cytolytic potential. For a more efficient assessment to be undertaken, mice that received the primary vaccination together with TSN1 were administered a.i. with the OVA antigen on d14 and T cells were isolated from the lung in 7 days (Figure 4A). CD8^+^ T cells that entered the lung were proven to be specific for the cognate antigen (Figure 4B). Flow cytometry analysis revealed a nearly two-fold increase in the recovery of pulmonary Tet^+^ CD8^+^ T cells in OPC + TSN1 mice compared to OPC-only controls in response to antigen invasion (Figure 4C). CXCR6 and CD49a, important for lung homing and adhesion, were increasingly expressed on Tet^+^ CD8^+^ T cells obtained from the lung of OPC + TSN1 mice (Figure 4D), suggesting a correlation between TSN1 and the increased recruitment of antigen-specific CD8^+^ T cells to the lung.

**Figure 5:**
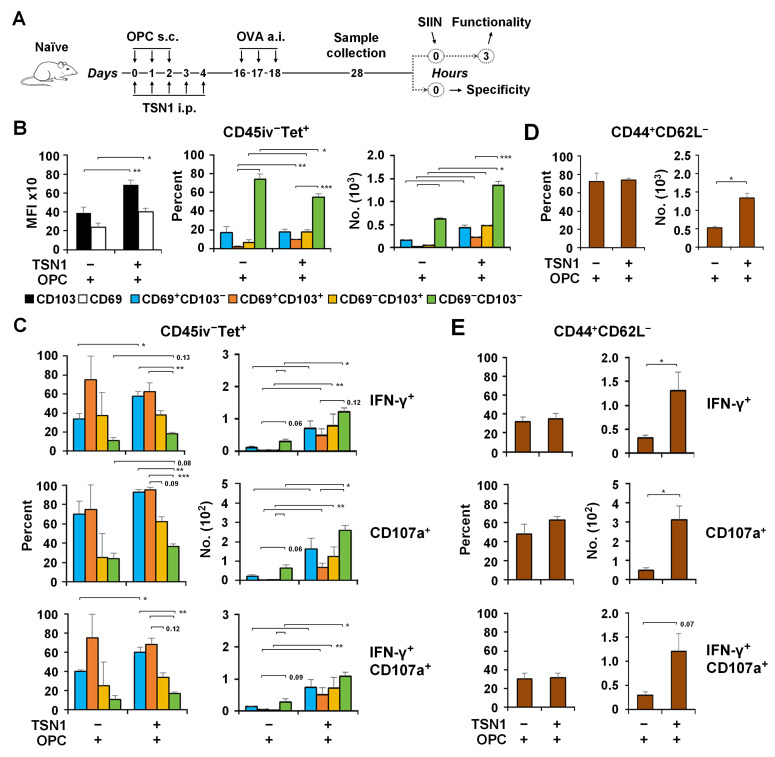
Subcutaneous immunization in combination with TSN1 enhances CD69^+^CD103^+^Trm differentiation. Mice were immunized s.c. by footpad injection of OPC while receiving 10 mg/kg bw/d of TSN1 given i.p. The OVA was delivered by a.i. at d14. Cells were isolated from the lung at d28 subsequent to i.v. labeling of CD45. Lungs were isolated and CD45iv^−^ Tet^+^ CD8^+^ T cells were gated for analysis. (**A**) Schematic representation of the immunization regimen. (**B**) MFI of CD69, CD103, and percentage and number of CD69^+^CD103^−^, CD69^+^CD103^+^, CD69^−^CD103^+^, and CD69^−^CD103^−^ cells in the lung. (**C**) Percentage and number of IFN-γ^+^, CD107a^+^, and IFN-γ^+^CD107a^+^ cells in each subset. (**D**) Percentage and number of CD44^+^CD62L^−^ cells in CD69^−^ Tet^+^CD8^+^ T cells. (**E**) Percentage and number of IFN-γ^+^, CD107a^+^, and IFN-γ^+^CD107a^+^ cells in CD44^+^CD62L^−^ cells. Results are expressed as mean ± SEM (*n* = 4–6 per group). Two-tailed Mann–Whitney test was used for analysis. * *p* < 0.05 and ** *p* < 0.01 and *** *p* < 0.001 indicate a significant difference between groups.

Dissection of the specific cell composition and function revealed that Tet^+^ CD8^+^ T cells in the lungs of OPC + TSN1 mice were negative for CD62L but expressed higher levels of the key Trm markers CD69, CD103, and CD44 than in OPC mice, indicative of enhanced tissue retention and an expanded ability to differentiate into Trm-like cells in the CD45iv^−^ population, more specifically the two CD69^+^ subsets, CD69^+^CD103^+^ and CD69^+^CD103^−^ (Figure 5A,B). Thus, TSN1 might play an important role in regulating cellular migration and tissue-resident memory differentiation of vaccine-induced CD8^+^ T cells. Further, more than half of the two CD69^+^ cells from OPC + TSN1 were able to produce IFN-γ and most expressed CD107a in response to antigen restimulation, while the other two subsets CD69^−^CD103^+^ and CD69^−^CD103^−^ had lower functionality (Figure 5C). OPC + TSN1 mice had a 7.7–17.5-fold increase in the number of IFN-γ^+^, CD107a^+^, and IFN-γ^+^CD107a^+^ cells for each subset compared to OPC mice, indicating the potent effect of TSN1 in retaining functional Trm. Although Tem, identified within the CD69^−^ cells, represented a significant population (Figure 5D), they had a decreased capacity of IFN-γ and CD107a production (Figure 5E), compared to Trm-like cells, 34.9% vs. 62.7%, *p* < 0.05 and 63.0% vs. 94.8%, *p* < 0.001, respectively. Therefore, TSN1 may also demonstrate an important role in promoting Tem to circulate through the lung and maintaining their functional ability, although not as effectively as Trm.

## 4. Discussion

**Figure 6:**
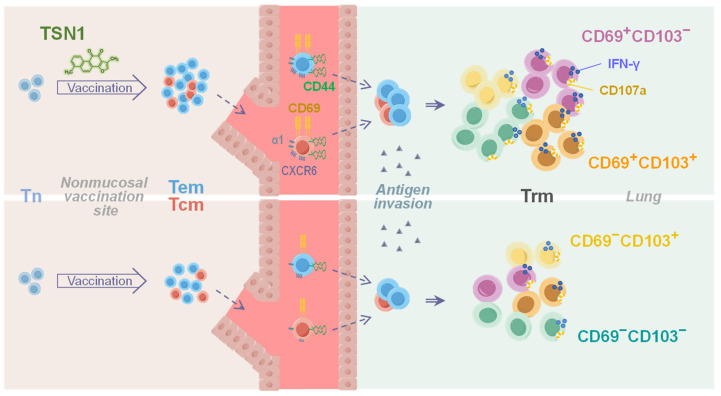
TSN1 administration during immunization helps the establishment of lung Trm with enhanced effector functions. Dashed line arrows: T cell trafficking; double-lined arrows: T cell subset differentiation.

Vaccination by a nonmucosal route to elicit cellular mucosal immunity against respiratory infections presents a great challenge for the development of an effective vaccine or immunization strategy. Previous studies employed mutant heat-labile enterotoxins from biological preparations as an adjuvant for intradermal vaccination of mice capable of stimulating more gut antigen-specific CD4^+^ T cells or pulmonary B cells, which are most likely tissue-resident [33,34]. Along with the impressive progress that has been achieved, there is still a need to identify some significant ways of improving nonmucosal vaccination effectiveness, such as utilizing natural products, to specifically elicit mucosal immunity. Our initial data results demonstrate that the parenteral, nonmucosal vaccination via the subcutaneous route effectively induces respiratory mucosal memory responses of CD8^+^ T cells that could likely be regulated and shaped by TSN1 during the priming phase due to its featured differential effects. The improved and robust formation of Tem and Tcm with additionally upregulated CD44, CD49a (α chain of VLA-1), and CD69 may facilitate transmigration of these antigen-specific memory subsets to the lung to establish tissue residency and exert effector functions when there is an injury to the tissue (Figure 6).

CD8^+^ Trm are well recognized for their immunological protection against pulmonary infection, providing prompt access to the infection site even before pathogenic immune evasion is fully operative. It is established that they comprise CD69^+^CD103^+^ in addition to two other memory subsets, CD69^+^CD103^−^ and CD69^−^CD103^−^, all of which are supposed to be equally divided within the memory CD8^+^ T cell group, preventing a reduction in memory T cell diversity or a decrease in the effectiveness of the secondary response [35]. The subsets that express CD69 and variable levels of CD103, namely CD69^+^CD103^±^, can arise in the lungs of healthy, infected or cancerous individuals and do not egress from the tissue [36,37,38,39]. Our study may also indicate that both CD103^−^ and CD103^+^ subsets of CD69^+^ Trm-like cells produce IFN-γ and express CD107a equally well and more effectively than CD69^−^CD103^−^ and CD69^−^CD103^+^ cells, suggesting an important role for CD69^+^ Trm-like cells in exerting the cytolytic effector function and probably successful adaptation of the immune system to fight off infections (Figure 6).

Indeed, CD103 plays an important role in the retention of Trm in the mucosal tissue and its expression is driven by TGF-β conceivably through suppression of TCF1 [40,41]. We demonstrate that a marked reduction in the percentage of CD69^−^CD103^−^ Trm occurs when TSN1 is included in the vaccination, allowing CD69^±^CD103^+^ to increase proportionally, highlighting the prominent role of TSN1 in driving the differentiation of CD103^+^ Trm. It could be argued that TSN1 may facilitate the formation of pulmonary CD103^+^ Trm by stimulating CD69^−^CD103^−^ cells as transiting Tem through differentiation or conversion can create a more efficient pool of tissue-bound memory T cells [38]. In fact, Tem and Trm share a common origin in the circulation and secondary lymphoid organs, with significant clonal overlap [12]. Trm that have trafficked to the tissue may originate from either of these sources through certain tissue-specific factors [42].

Moreover, Tcm could also give rise to Trm in response to viruses, tumors [43], or inflammation [44]. Tcm differentiate into Trm with a CD69^+^CD103^−^ phenotype following a recall response to virus challenge in an antigen-specific manner [45]. Inhibitors of T cell activation, specific lineages of dendritic cells, and integrins are believed to be involved in mediating the residency of T cells [43,44]. As demonstrated by this study, the increased frequency of cells relative to other subsets in mice treated with TSN1 may suggest the possibility for TSN1 to have a more direct role in initiating the differentiation of lung-homing Tcm into the subset with upregulated CD69 expression, since it displays the ability to upregulate CD69 on Tcm in vitro. Therefore, TSN1-facilitated Tem and Tcm contribute to the formation and localization of pulmonary Trm-like cells, more significantly CD69^+^CD103^+^, and to a lesser extent, CD69^+^CD103^−^, all with robust cytolytic potential. In consideration of the Trm heterogeneity and functionality implicated here as well as demonstrated in other tissues [46,47], future studies would expect to engage in a more in-depth exploration of how the phytocompound regulates pulmonary Trm differential capacity to improve tissue residency and local longevity and replenishment, and to mechanistically elucidate how these regulatory processes mediate effective mucosal defense and pathogen clearance, as well as the quality and durability of protection in the lung.

We observed that TSN1 exhibited bell-shaped or biphasic dose–response curves for Tem and Tcm, respectively, characterized by its capability to enable the expansion of Tem and, to a lesser extent, Tcm with low doses, and to inhibit their expansion with high doses but greater for Tcm than for Tem. This results in the formation of a fair amount of Tcm without substantially compromising the expansion of Tem, contrasting with what has been observed with berberine. Berberine is shown to be a potent anti-inflammatory agent [48] and causes a substantial, at least 3-fold reduction in the number of Tem from the initial level [26] with no expansion at all (unpublished results), while TSN1 is reported to have a rather weak anti-inflammatory effect [49]. The modest suppressive effect of TSN1 may facilitate Tem expansion along with Tcm development, leading to increased traffic through peripheral tissues and an enlarged population of CD8^+^ Trm cells [50].

## 5. Conclusions

To summarize, our data appear to show that nonmucosal vaccination with concurrent TSN1 administration is able to elicit an effective CD8^+^ T cell-mediated memory immune response in the respiratory mucosa. TSN1’s differential effect toward the formation of high-quality Tem and Tcm may facilitate the distribution of CD8^+^ T cells in peripheral tissue, more specifically to the lung, to differentiate into pulmonary Trm-like cells with functionally enhanced cytotoxic capacity that primarily engage the CD69^+^CD103^±^ as opposed to other subsets. As a significant constituent of *S. miltiorrhiza*, TSN1 may potentially serve as a novel immunomodulatory agent for the development of nonmucosal vaccination as an attractive alternative strategy to elicit mucosal immunity, although further research is required to confirm these preliminary findings. A case in point would be a situation in which TSN1 is incorporated in the development of a microbiota colonization strategy for immunization on the skin [51], where the epidermis is found to be essential for the induction of highly effective peripheral Tcm and Tem, leading to superior protective immunity in the skin and lungs [52]. Moreover, this natural compound may also be combined with or adapted to mucosal vaccine strategies [53], helping further establish and/or prolong the residency of memory T cells.

## Data Availability

The data presented in this study are available on request from the corresponding author. The data are not publicly available due to [the ongoing nature of the research but can be requested on reasonable request].
